# Determinants of syphilis infection among pregnant women attending antenatal care in hospitals of Wolaita zone, Southern Ethiopia, 2020

**DOI:** 10.1371/journal.pone.0269473

**Published:** 2022-06-03

**Authors:** Tigabu Addisu Lendado, Tessema Tekle, Desalegn Dawit, Wakgari Binu Daga, Chala Wegi Diro, Mihiretu Alemayehu Arba, Tadese Tekle

**Affiliations:** School of Public Health, College of Health Sciences and Medicine, Wolaita Sodo University, Wolaita Sodo, Ethiopia; Public Library of Science, UNITED KINGDOM

## Abstract

**Objective of the study:**

The objective of this study was to identify determinants of syphilis infection among pregnant women attending antenatal care in hospitals in the Wolaita zone, Southern Ethiopia,2020.

**Methods:**

An unmatched facility-based case-control study was conducted among pregnant women who received antenatal care at four randomly selected hospitals from September 1 to October 30, 2020. A two-stage sampling technique was used in the selection of hospitals and study participants. The data were collected from the participants using a pre-tested structured questionnaire and analyzed using STATA Release 15. Bivariate and multivariate logistic regression analyses were used to determine syphilis infection determinants. Crude and adjusted odds ratios were used for each explanatory variable with a 95% confidence level. A statistically significant association was declared when a p-value was less than 0.05.

**Results:**

A total of 296 (74 cases and 222 controls) pregnant women participated, with a recruitment rate of 97.4%. In multivariate logistic regression, the likelihood of developing a maternal syphilis infection was higher in pregnant women who had more than one-lifetime sexual partner [AOR = 3.59, 95% CI (1.09–11.71)]; a history of sexually transmitted infections [AOR = 3.46, 95%CI (1.32–9.08)] and used a substance [AOR = 3.39, 95%CI (1.31–8.77)].

**Conclusion:**

Sexual-related factors continued to be a major determinant of syphilis in pregnant women. The results suggest that there is a need to promote safe sexual behavior, raise awareness about the risk of STIs, and early diagnosis and treatment of STIs to control syphilis infection, and necessary to make the antenatal care service comprehensive for pregnant women.

## Background

Syphilis is one of the most common STIs (Sexual Transmitted Infections) which is caused by the spirochete *Treponema palladium* [1]. Around the world, 1 million or 0.8% of pregnancies are at risk of contracting syphilis. [2, 3]. Maternal syphilis infection is highly prevalent in Africa, accounting for 57% of the global burden [2]. This is also a public health problem in developing countries, like Ethiopia [4]. Various studies conducted in Ethiopia have shown that syphilis seroprevalence ranges from 4% to 15% in pregnant women receiving antenatal care [5–7]. Even with this high prevalence, syphilis testing in pregnant women is low in Africa, averaging 47 percent [2]. This low screening was a signal for those women who were not tested for syphilis.

Maternal syphilis infection is associated with various adverse pregnancy outcomes [8, 9]. It has contributed to over 143,000 fetal losses and stillbirths, 61,000 neonatal deaths, 44 000 preterm or low weight births, and 102 000 infected infants worldwide [2, 3]. It has had a very strong negative impact on sub-Saharan African countries. A study conducted in 43 countries in sub-Saharan Africa found that maternal syphilis infection caused the estimated incidence of approximately 205,901 adverse pregnancy outcomes per year. This contributes to an estimated 12.5 million Disability-Adjusted Life Years (DALY) [9]. Surprisingly, among these sub-Saharan countries, Ethiopia ranks in the top three because of the high number of syphilis-associated adverse pregnancy outcomes [9]. In addition, untreated pregnant women who were infected with syphilis were risky for congenital syphilis [10]. Nevertheless, these serious adverse pregnancy outcomes can be prevented and treated. Improving access to quality antenatal care for syphilis testing and early treatment for pregnant women would reduce the serious outcome of syphilis infection [8, 11, 12].

Syphilis can be passed on through sexual contact or between mother and child during pregnancy or delivery [10, 13]. Various studies have shown that various risk factors contribute to maternal syphilis. The factors responsible for maternal syphilis infection among pregnant women are multiple sexual partners, history of sexually transmitted infections, inconsistent condom use, illegal drug use, history of abortion, and a partner’s risky sexual behavior [6, 7, 14, 15].

Different strategies have been developed for reducing the burden of syphilis [13, 16, 17]. Global initiatives aimed to reduce the prevalence of syphilis by 90% and congenital syphilis to be less than 50 cases per 100,000 live births during the coming decade [13]. Reaching the targets can reduce the burden of maternal syphilis by 8.5 million DALYs [9]. The implementation of strategies and policies to address syphilis and its serious squeal in pregnant women has been influenced by complex and multi-faceted risk factors [18]. However, a limited number of studies have been conducted on the determinants of syphilis infection in pregnant women in Ethiopia, including in the study area. Therefore, this study sought to determine the determinants of syphilis infection in pregnant women attending ANC in hospitals in the Wolaita zone.

## Materials and methods

### Study design, period, and area

An unmatched case-control study was used to determine the determinants of syphilis infection among pregnant women in hospitals in the Wolaita Zone. The Wolaita zone is located 380Km from the capital of Addis Ababa and 157 km from the regional capital of Hawassa. Wolaita zone is situated at an altitude of 1500m-1800m above sea level [19]. The Wolaita zone consists of seven hospitals (one referral and teaching hospital, one general hospital, and five primary level hospitals). All hospitals provide antenatal care services, including syphilis screening for pregnant women. This study was carried out between September 1 and October 30, 2020.

### Study participants

Pregnant women aged 18+ were recruited to take part in the study. And those who were critically sick and unable to respond were excluded. Cases and controls were selected based on laboratory investigation. Pregnant women who were serologically positive for syphilis were selected as cases. And those that tested negative for serologic testing for syphilis were selected as controls.

### Sample size determination and sampling technique

The sample size is calculated based on Kelsey’s unmatched case-control formula using Open-Epi Version 303 software with the following assumptions: 95% confidence level, 80% power, the ratio of the case to control 1:3, percent of exposures among control = 4.63%, and AOR = 5.4. The percent of controls exposed and AOR was taken from the previous study that women who had more than a one-lifetime sex partner took as the main exposure variable for syphilis infection [20]. The sample size determined according to the above assumptions was 276 and, with an added non-response rate of 10%, it was 304. Based on the case-control ratio, the determined cases were 76, and the controls were 228.

**Fig 1:**
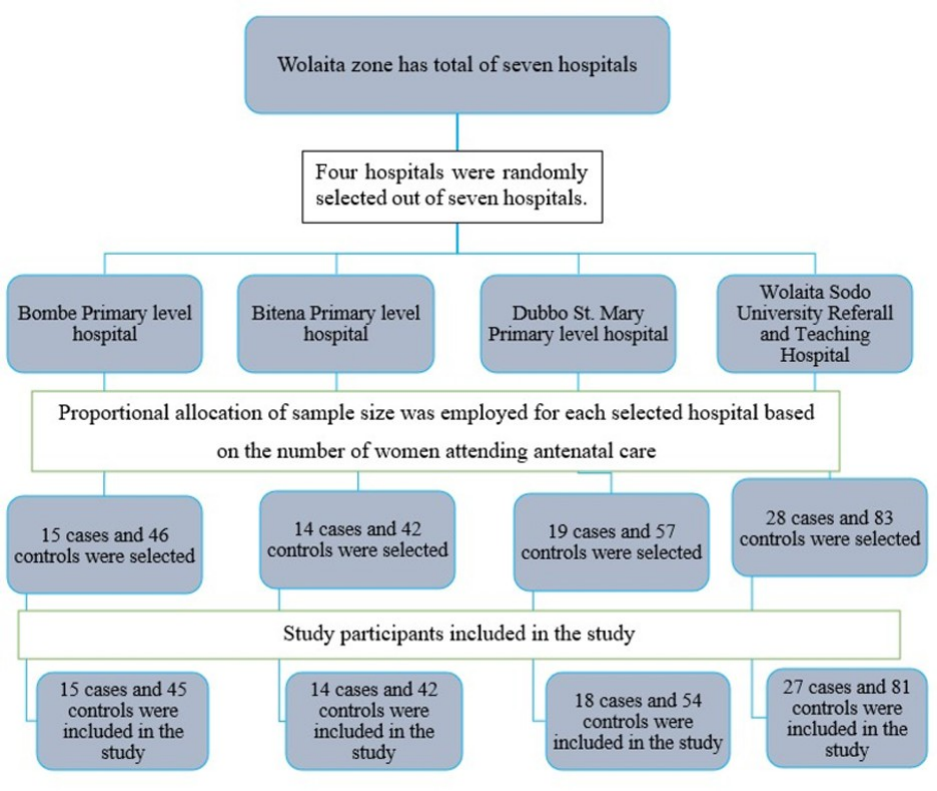
Schematic diagram for how the pregnant women were selected for a study of the determinants of syphilis infection among pregnant women attending antenatal care in hospitals of Wolaita Zone, 2020.

A two-stage sampling method was used to select the hospitals and study participants. Initially, hospitals were selected, followed by a selection of study subjects. Of the seven existing hospitals in the Wolaita Zone, four were selected using a lottery method. These are the Bitena primary level hospital, the Bombe primary level hospital, the Dubbo Saint Mary primary level hospital, and the Wolaita Sodo Referral and Teaching Hospital. Next, a proportional allocation of the sample size was used for each selected hospital according to the number of women receiving antenatal care. Finally, eligible pregnant women who are receiving antenatal care services at the hospital were recruited for the study until the required sample size was obtained (steps are described in Fig 1).

### Data collection instruments and techniques

Data was collected via the Open Data Kit (ODK) app installed on the smartphone. All smartphones used to collect data were Android’s 10th version, and their data connectivity was active throughout the data collection. No loss of data connection while collecting data gave up using the offline option. The data was collected by four qualified midwifery nurses and laboratory health professionals with experience in mobile data collection. Both midwifery nurses and laboratory health professionals have achieved an undergraduate degree and have completed higher education at a college or university. A supervisor was assigned to each hospital to ensure that the data was collected properly by data collectors and to address any problems they may have during data collection over the phone.

A structured questionnaire was used to collect the data, which was developed after reviewing various literature [6, 7, 14, 15, 21]. The questionnaire was broken down into three components. These include sociodemographic characteristics, factors related to the mother and partner, and awareness of syphilis prevention and treatment. It was prepared in English and translated into national (Amharic) and local (Wolaitegna) languages and translated back to English to keep consistent. We pre-tested this questionnaire. During the pre-test, the questionnaire was checked and corrected for irrelevant and unclear wording, the length of the questionnaire was appropriate, and the language used was easy to understand. In addition, it was tested to verify that it was appropriate for the analysis.

### Laboratory tests and procedures

Laboratory tests were performed using a serological test because it is currently the best method for testing and diagnosing syphilis [22]. Five milliliters (5ml) of blood were collected from a vein at the elbow for laboratory investigation. It was then collected in a test tube or vial to separate the serum from the whole blood by centrifuging at 3000 rpm for 5 minutes and transferred into labeled tubes.

**Fig 2:**
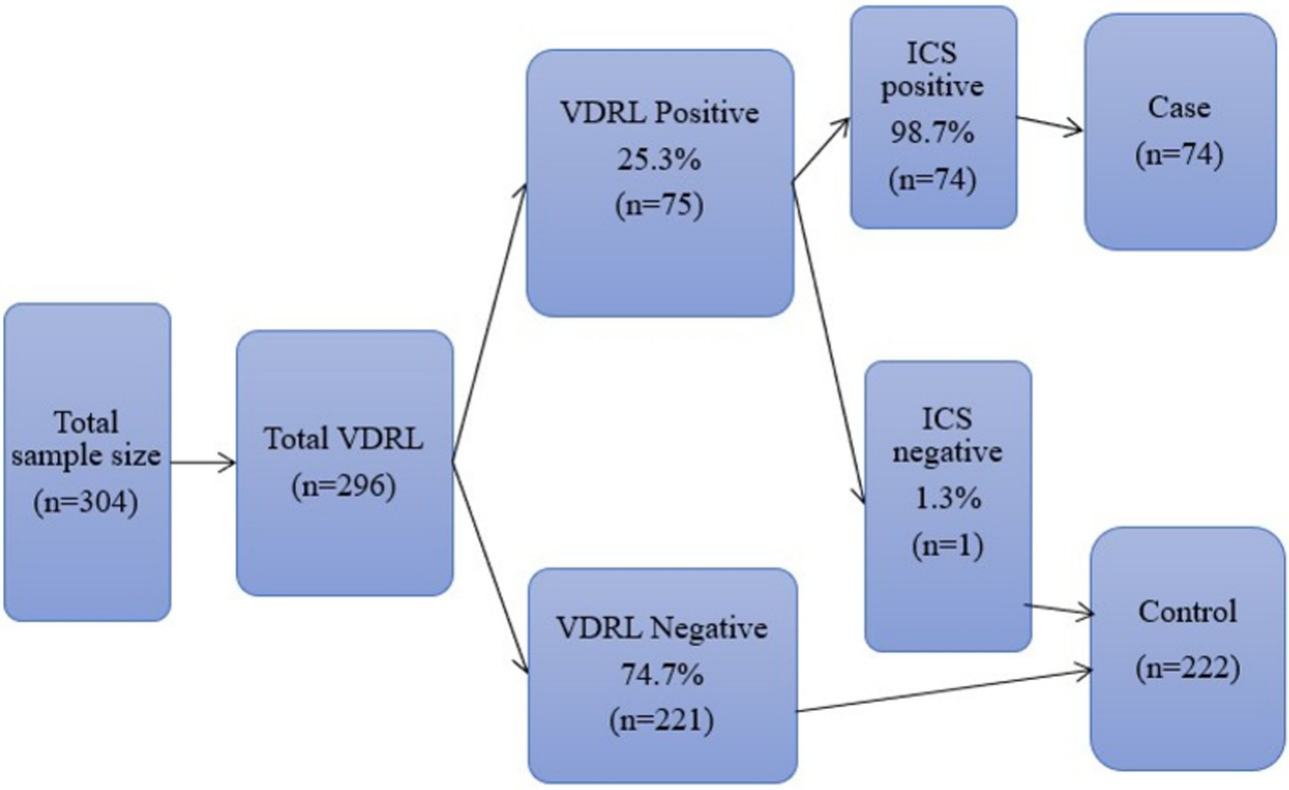
Laboratory testing procedure followed to ascertain cases and controls for the study of determinants of syphilis infection among pregnant women attending antenatal care in hospitals of Wolaita Zone, 2020.

Initially, the VDRL test was used to detect syphilis infection through the detection of non-Treponemal antibodies. Following this, women who tested positive for VDRL were subjected to the Immunochromatography test strips (ICS) for the qualitative detection of *T*. *palladium* antibodies. Both tests were aligned with the standard testing procedure. The ICS test has high sensitivity and specificity that would give a favorable result with standard tests (RPR/VDRL followed by FTA-Abs/TPHA) [23]. When VDRL and ICS tests were positive, women were found to be infected with syphilis (selected as cases). If not, the women were syphilis negative (selected as controls). There was strong agreement (98.7%) between the VDRL and ICS test results (Fig 2).

### Study variables

The outcome variable of the study was syphilis infection among pregnant women. The explanatory variables were (1) sociodemographic factors (age, marital status, educational status, partner educational status, occupation, partner occupation, residence), (2) maternal and partner-related factors (number of lifetime sexual partners, substance use, partner substance use, prior history of STIs, HIV, abortion, sexual coercion, blood transfusion, gravidity, and condom use), and awareness of syphilis prevention and treatment.

### Data quality

The training was provided to the data collectors for one day to assure data quality while collecting the data. The training focused on the objective of the study, the content of the questionnaire, the data collection procedure, the ODK execution, how to approach the respondents, and ethical issues such as patient confidentiality and privacy. Furthermore, the questionnaire was pre-tested at 5% (4 cases and 12 controls) of pregnant women before the main study in an unselected health facility (Bale Primary Hospital).

### Operational definition

#### Sexual coercion

Pregnant women who have any history of the act of forcing (or attempting to force) through physical body harm or any means to engage in sexual behavior against their will [24].

#### Substance use

Pregnant women who experience ever-use of commonly used substances in Ethiopia such as alcohol, khat, and tobacco [25, 26].

#### Awareness of syphilis prevention and treatment

The awareness about syphilis prevention and treatment was measured by eight items (see Table 3). The response was recoded as "1" if the women responded correctly and "0" if they did not respond correctly. Finally, the re-coded items were calculated and women who responded correctly to six or more items are considered to have a good awareness of syphilis prevention and treatment. And women who responded to fewer than six items correctly have poor awareness of syphilis prevention and treatment. This calculation method was described in the previously published paper and this was a frequently used method for quantification of awareness of syphilis prevention [27].

### Data processing and data analysis

Data collected by ODK version 1.28.4 was downloaded from a server in CSV format and imported to STATA version 15 computer software for analysis. For cases and controls, univariate analyses such as frequency, proportion, mean, and standard deviation were used to summarize the data. The Pearson Chi-square test was used to compare cases and controls with categorical variables. But we used Fischer’s exact test when the expected cell frequency was less than five. Before running regression, the correlation between explanatory variables was checked using the variance inflation factor (VIF). For syphilis determinants, bivariate and multivariate logistic regression analyses were used. Explanatory variables with a p-value lower than 0.20 in bivariate logistic regression were input into the multivariate logistic regression to control potential confounders. The determinants of syphilis infection were known from multivariate logistic regression analysis with a p-value less than 0.05 and adjusted odds ratios (AORs) with a 95% CI. The final logistic model was tested for fit using the Hosmer and Lemeshow test (p-value = 0.30). Finally, the results are summarized in the form of texts and tables.

### Ethics approval and consent to participate

The study was ethically approved by the Ethical Review Committee (ERC) of Wolaita Sodo University. All methods used conformed to the ethical principle of the World Medical Association Declaration of Helsinki for research on human subjects. Informed consent was provided by the participants. It was obtained from their legal guardian/LAR for those who do not read and write. And to remain anonymous, the name of the participants was not included in the study. Participation is voluntary, and participants who are unwilling to continue the interview may withdraw from the study at any desired time has been ensured during data collection. Pregnant women who tested positive for syphilis infection were treated in the hospital, and those who tested positive for HIV were linked to an anti-retroviral treatment center.

## Results

### Socio-demographic characteristics

A total of 296 (74 cases and 222 controls) pregnant women participated, resulting in a recruitment rate of 97.4%. The mean age of pregnant women in cases and controls was 27.82 ± 5.57 and 27.44 ± 4.70 years respectively. There were 16.22% of pregnant women who have achieved a diploma and a higher level of education had syphilis. Nearly all (98.65%) of pregnant women who had syphilis were married, and 35 (47.3%) of pregnant women who had syphilis were housewives. About 49 (66.2%) and 152 (68.5%) of urban pregnant women had and had not been infected with syphilis, respectively (see Table 1).

**Table 1:** Sociodemographic characteristics of study participants for syphilis infection among pregnant women in hospitals of Wolaita zone, 2020.

Variables		Cases (n = 74)	Controls (n = 222)	Total n = 296	χ^2^	P-value
		n (%)	n (%)	n (%)		
Age	15–24	19 (25.7)	58 (26.13)	77 (26)	2.34	0.31
	25–34	45 (60.8)	147 (66.21)	192 (64.9)		
	35–49	10 (13.5)	17 (7.66)	27 (9.1)		
Marital status	Married	73 (98.65)	216 (97.3)	289(97.6)	0.44	0.68
	Non-married	1 (1.35)	6 (2.7)	7 (2.4)		
Educational status	Can’t read and write	8 (10.81)	12 (5.4)	20 (6.8)	6.27	0.18
	Read and write	24 (32.43)	89 (40.1)	113 (38.2)		
	Primary education	11 (14.87)	19 (8.56)	30 (10.1)		
	Secondary education	19(25.67)	54 (24.32)	73 (24.7)		
	Diploma and above	12 (16.22)	48 (21.62)	60 (20.3)		
Partner educational status	Can’t read and write	8 (10.8)	9 (4)	19 (5.7)	7.44	0.11
	Read and write	28 (37.84)	90 (40.5)	117 (39.9)		
	Primary education	5 (6.76)	17 (7.7)	22 (7.4)		
	Secondary education	13 (17.6)	25 (11.3)	38 (12.8)		
	Diploma and above	20 (27)	81 (36.5)	100 (34.1)		
Occupation	Employed	12 (16.2)	50 (22.5)	62 (20.9)	7.54	0.10
	Merchant	14 (18.9)	63 (28.4)	77 (26.0)		
	Housewife	35 (47.3)	85 (38.3)	120 (40.5)		
	Daily laborer	8 (10.8)	10 (4.5)	18 (6.1)		
	Student	5 (6.75)	14 (6.3)	19 (6.4)		
Partner occupation	Employed	17 (23)	84 (37.84)	101 (34.1)	6.77	0.15
	Merchant	35 (47.3)	75 (33.8)	110 (37.2)		
	Farmer	12 (16.2)	34 (15.3)	46 (15.5)		
	Daily laborer	6 (8.1)	14 (6.3)	20 (6.8)		
	Student	4 (5.4)	15 (6.76)	19 (6.4)		
Residence	Urban	49 (66.2)	152 (68.5)	200 (67.9)	0.13	0.72
	Rural	25 (33.8)	70 (31.5)	96 (32.1)		

n, number of participants included in the study

### Maternal and partner-related factors

Twelve (14.3%) of pregnant women who had more than one-lifetime sex partner had syphilis, while only seven (3.2%) had no syphilis. Seventeen (23%) of pregnant women who had syphilis used a substance, and twenty-two 22 (29.7%) of pregnant women who had syphilis had a partner that used a substance. About 10 (13.5%) of pregnant women with a history of blood transfusion became infected with syphilis. An estimated 18 (24.3%) syphilis were reported in pregnant women with previous STIs (see Table 2).

**Table 2:** Maternal and partner-related characteristics of syphilis infection among pregnant women in hospitals of Wolaita zone, 2020.

Variables		Cases (n = 74)	Controls (n = 222)	Total (n = 296)	χ^2^	P-value
Category						
		n (%)	n (%)	n (%)		
Number of lifetime sex partners	1	62 (83.7)	215 (96.8)	277 (93.6)	15.77	<0.001
	>1	12 (14.3)	7 (3.2)	19 (6.4)		
Substance use	Yes	17 (23)	18 (8.1)	35 (11.8)	11.76	0.001
	No	57 (77)	204 (91.9)	266 (88.2)		
Partner substance use	Yes	22(29.7)	46 (20.7)	68 (23.0)	2.54	0.11
	No	52 (70.3)	176 (79.3)	228 (77.0)		
HIV testing	Yes	54 (73)	160 (72)	214 (72.3)	0.02	0.88
	No	20 (27)	62 (28)	82 (27.7)		
Blood transfusion	Yes	10 (13.5)	14 (6.3)	24 (8.1)	3.87	0.05
	No	64 (86.5)	208 (93.7)	272 (91.9)		
Abortion	Yes	15 (20.2)	19 (8.5)	34 (11.5)	7.49	0.006
	No	59 (79.8)	203 (91.5)	262 (88.5)		
Coerced	Yes	4 (5.4)	4 (1.8)	8 (2.7)	2.74	0.11
	No	70 (94.6)	218 (98.2)	288 (97.3)		
STI	Yes	18 (24.3)	16 (7.2)	34 (11.5)	15.99	<0.001
	No	56 (75.7)	206 (92.8)	262 (89.5)		
Gravidity	1–4	68 (91.2)	209 (94.1)	277 (93.6)	0.47	0.49
	> = 5	6 (8.8)	13 (5.9)	`19 (6.4)		
Condom use	Never use	72 (97.3)	203 (91.4)	275 (92.9)	2.88	0.89
	Use	2 (2.7)	19 (8.6)	21 (7.1)		

n, number of participants included in the study, HIV, Human Immunodeficiency Virus

### Awareness of syphilis prevention and treatment

Over half of the study participants (56.7%) had poor awareness about the prevention and treatment of syphilis in pregnant women with syphilis. While 32 (43.3%) of the study participants had a good awareness about the prevention and treatment of syphilis in pregnant women with syphilis (see Table 3).

**Table 3:** Description of items used for assessing awareness of syphilis prevention and treatment in the hospital of Wolaita zone, 2020.

Items used for assessing awareness of syphilis prevention and treatment		Cases (n = 74)	Controls (n = 222)	Total (n = 296)	χ^2^	P-value
		n (%)	n (%)	n (%)		
Item 1: Syphilis is mainly transmitted through sexual contact	Yes	68 (91.2)	208 (93.7)	276 (93.2)	0.28	0.59
	No	6 (8.8)	14 (6.3)	20 (6.8)		
Item 2: Syphilis is curable (if it is treated)	Yes	69 (93.2)	199 (89.6)	268 (90.5)	0.84	0.36
	No	5 (6.8)	23 (10.4)	28 (9.5)		
Item 3: A man or woman looks healthy may have syphilis	Yes	61 (82.4)	184 (82.9)	245 (82.8)	0.01	0.93
	No	13 (17.6)	38 (17.1)	51 (17.2)		
Item 4: Using condoms correctly in sexual contact can prevent syphilis transmission	Yes	53 (71.6)	164 (73.8)	217 (73.3)	0.14	0.70
	No	21 (28.4)	58 (26.2)	79 (26.7)		
Item 5: Syphilis infection can increase the risk of HIV transmission or acquisition	Yes	46 (61.2)	147 (66.2)	193 (65.2)	0.40	0.53
	No	28 (37.8)	75 (33.8)	103 (34.8)		
Item 6: Sex partners of syphilis patients need to attend a hospital for serological examination	Yes	52 (70.3)	199 (89.6)	251 (84.8)	16.15	<0.001
	No	22 (29.7)	23 (10.4)	45 (15.2)		
Item 7: Syphilis infected women can transmit syphilis to their neonatal	Yes	40 (54.1)	133 (60)	173 (58.4)	0.78	0.38
	No	34 (45.9)	89 (40)	123 (41.6)		
Item 8: Having dinner or shaking hands with syphilis patients can infect syphilis	Yes	30 (40.5)	65 (29.3)	95 (32.1)	3.23	0.07
	No	44 (59.5)	157 (70.7)	201 (67.9)		
Awareness of syphilis prevention and treatment	Poor	42 (56.7)	97 (43.7)	139 (47.0)	3.80	0.05
	Good	32 (43.3)	125 (56.3)	157 (53.0)		

n, number of participants included in the study

### Factors associated with maternal syphilis

With a bivariate logistic regression: maternal education, maternal occupation, partner occupation, lifetime sexual partner, history of STIs, abortion, and substance use were significantly associated with syphilis among pregnant women. After controlling for confounding factors in multivariate logistic regression, lifetime sexual partner, STI history, and substance use were identified as determinants of syphilis infection among pregnant women. Pregnant women who had more than one lifetime sexual partner were 3.59 times [AOR = 3.59, 95% CI (1.09–11.71)] more likely to become infected with syphilis. The likelihood of syphilis was more than three times [AOR = 3.39, 95%CI (1.31–8.77)] in pregnant women using a substance. The pregnant women who had a history of STIs were 3.46 times [AOR = 3.46, 95%CI (1.32–9.08)] more likely to develop syphilis than women who had no previous history (see Table 4).

**Table 4:** Bivariate and multivariate logistic regression to determine determinants of syphilis infection among pregnant women attending ANC in hospitals of Wolaita zone, 2020.

Variables	Category	COR (95%CI)	P-value	AOR (95%CI)
Age of mothers	15–24	1		1
	25–34	0.81 (0.43–1.56)	0.54	0.87 (0.40–1.88)
	35–49	2.07 (0.80–5.36)	0.13	1.58 (0.45–5.53)
Educational status	Can’t read and write	1		1
	Read and write	0.40 (0.15–1.10)	0.08	0.54 (0.14–2.01)
	Primary education	0.87 (0.27–2.78)	0.81	0.58 (0.12–2.79)
	Secondary education	0.58 (0.19–1.49)	0.23	0.43 (0.09–1.95)
	Diploma and above	0.37 (0.13–1.12)	0.08	0.37 (0.05–2.45)
Partner educational status	Can’t read and write	1		1
	Read and write	0.35 (0.12–0.99)	0.048	0.31 (0.07–1.37)
	Primary education	0.33 (0.08–1.31)	0.12	0.54 (0.09–3.00)
	Secondary education	0.58 (0.18–1.87)	0.37	0.65 (0.12–3.45)
	Diploma and above	0.28 (0.09–1.81)	0.02	0.49 (0.10–2.45)
Occupation	Employed	1		1
	Merchant	0.93 (0.39–2.18)	0.86	0.54 (0.14–2.17)
	Housewife	1.71 (0.82–3.60)	0.15	0.86 (0.22–3.37)
	Daily laborer	3.33 (1.08–10.24)	0.04	1.79 (0.34–9.54)
	Student	1.49 (0.45–4.94)	0.52	1.03 (0.22–4.82)
Partner occupation	Employed	1		1
	Merchant	2.30 (1.19–4.45)	0.02	2.56 (0.75–8.74)
	Farmer	1.74 (0.75–4.04)	0.19	0.85 (0.21–3.48)
	Daily laborer	2.12 (0.71–6.29)	0.18	1.77 (0.37–8.42)
	Student	1.32 (0.39–4.46)	0.66	1.38 (0.32–5.89)
Life-time sex partner	One	1		1
	Greater than one	5.94 (2.24–15.74)	<0.001	**3.59 (1.09–11.71)** *
Substance use	Yes	3.38 (1.64–6.98)	0.001	**3.39 (1.31–8.77)** *
	No	1		1
Partner substance use	Yes	1.62 (0.89–2.93)	0.11	1.19 (0.49–2.89)
	No	1		1
Blood transfusion	Yes	2.32 (0.98–5.48)	0.06	2.14 (0.79–5.83)
	No	1		1
Abortion history	Yes	2.72 (1.30–5.67)	0.008	1.22 (0.44–3.37)
	No	1		1
STIs history	Yes	4.14 (1.98–8.63)	<0.001	**3.46 (1.32–9.08)** *
	No	1		1
Coerce history	Yes	3.11 (0.76–12.78)	0.11	1.35 (0.21–8.83)
	No	1		1
Awareness of syphilis prevention and treatment	Poor awareness	1.69 (0.99–2.87)	0.05	1.53 (0.79–2.96)
	Good awareness	1		1
Condom use	Yes	1	0.11	1
	No	3.37 (0.77–14.83)		2.81 (0.50–15.70)

COR, Crude Odd Ratio; AOR, Adjusted Odd Ratio; STI, Sexual Transmitted Infection.

*Indicates p-value less than 0.05 in multivariate logistic regression

## Discussion

This study sought to identify the determinants of syphilis infection in pregnant women receiving antenatal care services. The number of lifetime sexual partners, a history of STIs, and a history of substance use were identified as determinants of syphilis infection in pregnant women. The likelihood of syphilis infection in pregnant women was higher for women who had more than one lifetime sexual partner than for those who had an only one-lifetime sexual partner. This was supported by a study in South Gondar and Jimma that found that syphilis was associated with multiple sexual partners [6, 28]. Engaging in sexual relations with multiple partners exposes the women to an increased risk of STIs [29, 30]. Furthermore, individuals with multiple sexual partners have a higher risk of engaging in unprotected sex [31]. This, in turn, may increase the risk of syphilis infection [32]. The practice of multiple sex partners is now becoming a public health issue in Ethiopia [30]. Therefore, we suggest promoting safer sexual behaviors such as the use of condoms and incorporating the service into routine antenatal care to improve the health of the woman and unborn child [33].

This study found that pregnant women with a history of sexually transmitted infections had a higher risk of developing syphilis. This finding was also supported by a study indicating that pregnant women with a history of STIs were more likely to develop syphilis than those who did not [32]. STIs can cause a breakup or sore skin, which can facilitate the transmission of syphilis during sex [34]. Yet STI continues to be a major public health challenge in the African Region and around the world [35]. There is still a need to educate women about their risk of STIs, increase the use of existing barrier methods during intercourse, and develop new methods of protection against STIs [36, 37]. The STI strategy proposed the continuum of services through prevention, diagnosis, treatment, and follow-up of women infected with STIs were suggested to reduce the burden of syphilis [11, 17, 38].

In this study, syphilis was more likely to occur in pregnant women with a history of substance use. Studies carried out in developed countries supported this finding [39–41]. In 2016, the US spent $442 billion on drug and alcohol use [42]. Furthermore, substance use has become a public health problem in Ethiopia today, despite the national master plan on drug control [43]. People with substance use disorder have an increased risk of engaging in risky sexual behaviors, exposing them to a higher risk for sexually transmitted diseases [44]. As well, pregnant women with a history of substance or drug use were reluctant to seek antenatal care and to be tested for and treated for syphilis. They have multiple special needs that require multi-professional treatment covering aspects of medical, psychological, and social attention. Unfortunately, there was no universally accepted guideline existed for the treatment of pregnant women who use substances [45]. This study could indicate that decision-makers must look for the best strategies that will benefit them. We recommend for health care professionals of treating substance-using women in both pharmacological and non-pharmacological intervention to enable them to use the clinical services that are essential to mothers and newborns.

This study has its strength and limitation. The strength of this study was a selection of both cases and controls from the same source that has its part to minimize selection bias. In addition, the case-control ratio (1:3) is important for improving the statistical efficiency of the study. However, this study is subject to potential recall and social desirability bias, with a few questions related to stigma and difficult to remember. As well, it is necessary to consider the limitation of the laboratory test for syphilis detection and diagnosis.

## Conclusion

Sexual-related factors are still the major determinants of syphilis infection in pregnant women. Substance use is also becoming a public health problem that leads women to syphilis. Therefore, the results suggest that there is a need to raise awareness about their risk of STIs and promote safe sexual behavior with their partners. Early diagnosis and treatment of STIs in pregnant women would be an important intervention to curb syphilis infection in pregnant women and stop its sequel to the unborn child [17, 37]. In addition, women who use a substance need to be treated and addressed in a holistic way [38, 45].

## Supporting information

S1 Dataset (DTA)

## Abbreviations

ANC: Antenatal Care
AOR: Adjusted Odd Ratio
ART: Anti-Retroviral Therapy
CI: Confidence Interval
CSV: Comma Separated Value
FTA-Abs: Fluorescent Treponemal Antibody Absorption Test
ICS: Immunochromatographic strip
ODK: Open Data Kit
SPSS: Statistical Software for Social Science
STI: Sexually transmitted infection
VDRL: Venereal Disease Research Laboratory
WHO: World Health Organization
